# Pbrm1 intrinsically controls the development and effector differentiation of iNKT cells

**DOI:** 10.1111/jcmm.17445

**Published:** 2022-06-29

**Authors:** Xin Wang, Lei Lei, Yanhong Su, Jun Liu, Ning Yuan, Yang Gao, Xiaofeng Yang, Chenming Sun, Bin Ning, Baojun Zhang

**Affiliations:** ^1^ Department of Pathogenic Microbiology and Immunology, School of Basic Medical Sciences Xi'an Jiaotong University Xi'an Shaanxi China; ^2^ Institute of Infection and Immunity, Translational Medicine Institute Xi'an Jiaotong University Health Science Center Xi'an Shaanxi China; ^3^ Xi'an Key Laboratory of Immune Related Diseases Xi'an Shaanxi China; ^4^ Key Laboratory of Environment and Genes Related to Diseases (Xi'an Jiaotong University), Ministry of Education Xi'an Shaanxi China; ^5^ Department of Kidney Transplantation, Nephropathy Hospital The First Affiliated Hospital of Xi'an Jiaotong University Xi'an China; ^6^ Jinan Central Hospital Shandong University Jinan Shandong China

**Keywords:** development, differentiation, iNKT cells, Pbrm1, RORγt

## Abstract

Under static condition, the pool size of peripheral invariant natural killer T (iNKT) cells is determined by their homeostatic proliferation, survival and thymic input. However, the underlying mechanism is not fully understood. In the present study, we found that the percentage and number of iNKT cells were significantly reduced in the spleen, but not in the thymus of mice with deletion of polybromo‐1 (Pbrm1) compared to wild type (WT) mice. Pbrm1 deletion did not affect iNKT cell proliferation and survival, instead significantly impaired their development from stage 1 to stage 2. Importantly, loss of Pbrm1 led to a dysfunction of RORγt expression and iNKT17 cell differentiation, but not iNKT1 and iNKT2 proportion. Collectively, our study reveals a novel mechanism of Pbrm1 controlling the peripheral size of iNKT cells through regulating their development and differentiation.

## 1 INTRODUCTION

As an “innate‐like” T cells, invariant natural killer T (iNKT) cells express both T cell marker (CD3) and NK cell marker (NK1.1).^1^, ^2^, ^3^ iNKT cells play a crucial role in eliminating bacterial, viral and fungal infection through recognizing glycolipids presented by the nonclassical MHC molecule CD1d.^4^, ^5^, ^6^, ^7^, ^8^, ^9^, ^10^ iNKT cell development originates from CD4^+^CD8^+^ double‐positive (DP) thymocytes, undergo T cells receptor (TCR) rearrangement and selection.^11^, ^12^, ^13^, ^14^ Under precise modulation, iNKT cell development in the thymus is divided into 4 stages: CD24^hi^CD44^lo^NK1.1^−^ (stage 0), CD24^lo^CD44^lo^NK1.1^−^ (stage 1), CD24^lo^CD44^hi^NK1.1^−^ (stage 2), and CD24^lo^CD44^hi^NK1.1^+^ (stage 3).^3^, ^7^ A number of transcription factors govern the development of iNKT cells, promoting iNKT cells maturation, protecting them from apoptosis, and stimulating thymocytes proliferation. As a key transcriptional regulator, promyelocytic leukemia zinc finger protein (PLZF) expression is upregulated specifically in stage 1 and 2 iNKT cells, but significantly decreased in the stage 3. Indeed, abrogation of PLZF leads to a developmental block in stage 0 of iNKT cells.^15^ Along with the iNKT cell development, Egr1/2 mediates the transition from stage 1 to stage 2 through binding to the promoter of PLZF and regulating its transcription.^16^ Whereas Notch1/2 signaling is involved in the progression to stage 3, as loss of Notch1/2 leads to a decreased proportion of iNKT cells at stage 3.^17^ Besides, E protein,^18^ Hobit^19^ and T‐bet^20^, ^21^ also contribute to the regulation of the stage 3 of iNKT cell development. Additionally, several other transcription factors are involved in iNKT cell survival such as c‐Myc,^22^ NF‐κB^23^ and TCF‐1.^24^

Based on the expression of master transcription factors, mature iNKT cells can be categorized into three functional subsets, namely iNKT1, iNKT2 and iNKT17.^25^ PLZF, with varied expression level among iNKT subsets, is also essential for their functional differentiation.^15^ Compared to iNKT2 cells, which express the largest amount of PLZF, IFN‐γ‐secreting iNKT1 cells express much lower amount of PLZF. Whereas, the expression level of PLZF in iNKT17 cells is between iNKT1 and iNKT2 cells.^26^ As the traditional T helper cell‐specific transcription factors, T‐box 21 (T‐bet), GATA binding protein‐3 (GATA3) and retinoic acid receptor‐related orphan nuclear receptor gamma (RORγt), also regulate the iNKT lineage decisions.^27^, ^28^, ^29^, ^30^ Loss of T‐bet exhibits a block in the differentiation of iNKT1 along with decreased interferon‐γ (IFN‐γ) production.^21^ Furthermore, GATA3 expression is essential for iNKT2 cells differentiation and IL‐4 production,^28^ while RORγt,^29^, ^30^ combined with other factors like E protein,^18^ is involved in iNKT17 cell differentiation. Nevertheless, there are still largely unknown regulators for iNKT cell development in the thymus and their effector differentiation in the periphery.

Pbrm1, as a subunit of the Pbrm1‐Brg1/Brm‐associated factors (PBAF) complex, interacts with ARID2, BRD7, BAF45A, as well as several other subunits to form a SWI/SNF chromatin remodeling complex.^31^, ^32^, ^33^ As a tumor suppressor, Pbrm1 is mutated frequently in diverse cancer types, and its reduced expression is positively correlated with the tumor progression.^34^, ^35^, ^36^, ^37^ Pbrm1 has also been reported to be involved in regulating T cell development and differentiation. Although Pbrm1 deficiency did not strongly affect the thymocyte development and the proportion of peripheral T cells,^38^ loss of Brg1 exhibited a delay of CD8 expression in DP cells, suggesting a favorable role for CD8 transcription.^39^, ^40^ In addition, Brg1 is required for T helper differentiation including Th1^41^ and Th2^42^ since loss of Brg1 caused a reduction in both IFNγ and IL‐4 production under the polarized condition. Other studies reported that Pbrm1 is a repressor of IL‐10 transcription in Th2 cells by either binding directly to the regulatory region in the Il‐10 locus, or regulating histone acetylation and CBP recruitment to the IL‐10 locus.^38^ A recent study also showed that Pbrm1 deletion caused a downregulation of foxp3 expression and an impairment in Treg suppressive function.^32^ Yet, the function of Pbrm1 in other lineage of immune cells is largely unknown.

In the present study, by utilizing mice with T‐cell‐specific ablation of Pbrm1 and *in vivo* adoptive transfer model, we demonstrated that Pbrm1 deficiency did not affect CD4^+^ and CD8^+^ T cell proportions in the thymus and spleen. However, Pbrm1‐deficient iNKT cells were significantly reduced in the spleen, as Pbrm1 specifically blocked the transition of iNKT cell development from stage 1 to stage 2 in the thymus. Furthermore, Pbrm1‐deficient mice exhibited an impairment of RORγt expression and iNKT17 lineage differentiation. Therefore, our study reveals a novel role of Pbrm1 in regulating the development and differentiation of iNKT cells.

## 2 MATERIAL AND METHODS

### 2.1 Mice

The Pbrm1^f/f^ strain was purchased from The Jackson Laboratory. Pbrm1 KO mice (LckCre^+^Pbrm1^f/f^) were generated by crossing Pbrm1^f/f^ mice with LckCre transgenic strain. Eight‐week‐old Lckcre^+^Pbrm1^+/+^ (WT) and Lckcre^+^Pbrm1^f/f^ (KO) male mice were analyzed in these experiments. All mice were bred and maintained in the specific pathogen‐free conditions by Xi'an Jiaotong University Division of Laboratory Animal Research. All the procedures were approved by the Institutional Animal Care and Use Committee of Xi'an Jiaotong University.

### 2.2 Antibodies and reagents

The Abs used are as follows: APC/Cy7 anti‐mouse CD4 (clone GK1.5), FITC anti‐mouse CD8a (clone 53–6.7), FITC anti‐mouse CD3 (clone 17A2), Pacific Blue™ anti‐mouse CD3 (clone 17A2), PE 45668 mCD1d, APC 42410 mCD1d, PE anti‐mouse PLZF(clone 9E12), FITC anti‐BrdU (clone 3D4), Alexa Fluor® 647 anti‐Brdu (clone 3D4), PE anti‐Annexin V (Cat # 640947), PE/Cy5 anti‐mouse CD19 (clone 6D5), PE/Cy5 anti‐mouse/human CD11b (clone M1/70), PE/Cy5 anti‐mouse CD11c (clone N418), APC/Cy7 anti‐mouse CD45.1 (clone A20), FITC anti‐mouse CD45.2 (clone 104), Fixation Buffer(Cat # 420801) and Intracellular Staining Perm Wash Buffer(Cat # 421002). All reagents were purchased from BioLegend (San Diego, CA, USA). PE/Cy7 anti‐Mo RORγt (clone B2D), PE anti‐Mo/Rt Ki‐67 (clone SolA15), Transcription Factor Fixation/Permeabilization Concentrate and Diluent were purchased from eBioscience. The APC BrdU Flow Kit was purchased from BD Biosciences.

### 2.3 Flow cytometry

Single cells were obtained from the thymi and spleens of indicated mice. For cell surface analysis, a total of 1–5 × 10^6^ cells were stained with indicated Abs in the dark at 4 C for 30 min. After washing with cold FACS buffer (1 × PBS supplemented with 2% FBS), cells were analyzed using CytoFLEX flow cytometer (Beckman Coulter, Brea, CA, USA). Flowjo software (CytExpert) was used for data analysis.

For cell proliferation analysis in vivo, 100 μl (10 mg/ml) of BrdU was injected intraperitoneally per mouse 4 h before sacrifice. Single cells were obtained from the spleens of indicated mice and treated according to FITC BrdU Flow Kit (BD Biosciences) or Ki67 antibody staining, and then were performed with FACS analysis.

For apoptosis analysis, freshly‐isolated cells were stained with the antibodies for indicated surface markers. After wash with FACS buffer, apoptosis was evaluated with Annexin V as previously described.^43^

To analyze intracellular transcriptional factors, after a 30 min surface staining, cells were fixed and permeabilized according to the manual of the Foxp3 kit, followed by anti‐RORγt and anti‐PLZF antibody staining and FACS analysis.

### 2.4 Bone marrow chimera

Bone marrow cells from WT (CD45.2^+^) and Pbrm1 KO mice (CD45.2^+^) were isolated and stained with lineage antibodies (anti‐CD4, CD8, CD3, CD19, CD11b, CD11c and TER‐119). The progenitor cells (lineage negative) were sorted by BD FACSAria™ II cell sorter (BD Biosciences, San Jose, CA, USA). Purified cells were adoptively transferred intravenously into age‐ and sex‐matched, lethally irradiated (7.5 Gy) WT mice (CD45.1^+^). Donor thymocytes and splenocytes were analyzed by flow cytometry 7 weeks after cell transfer.

### 2.5 Statistical analysis

Statistical analysis was applied to biologically independent mice or technical replicates for each experiment. Each experiment was independently repeated for three times. The two‐tailed Student's t test was used for all statistical calculations using GraphPad Prism 7 software. The level of significance is indicated as *p < 0.05, **p < 0.01, ***p < 0.001, ****p < 0.0001.

## 3 RESULTS

### 3.1 A reduction of peripheral iNKT cells in Pbrm1 deficient mice

**FIGURE 1:**
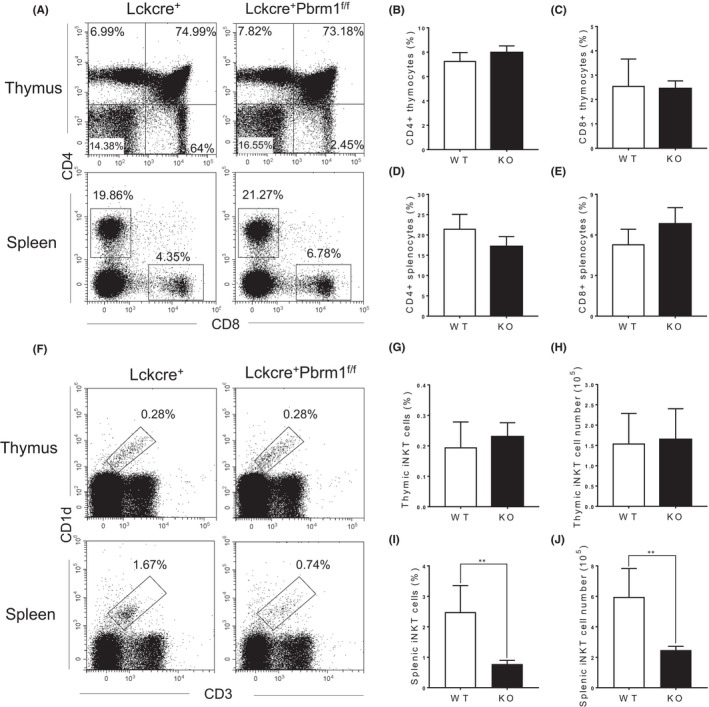
Reduced proportion of iNKT cells in the periphery of Pbrm1 deficient mice. Eight‐week‐old Lckcre^+^Pbrm1^+/+^ (WT) and Lckcre^+^Pbrm1^f/f^ (KO) mice were analyzed for CD4^+^ T cells, CD8^+^ T cells and iNKT cells by flow cytometry (*n* = 3). (A) Representative FACS plots of CD4^+^ and CD8^+^ T cells in the thymi and spleens from WT and Pbrm1 KO mice. (B, C) Statistical percentages of CD4^+^ and CD8^+^ thymocytes. (D, E) Statistical percentages of CD4^+^ and CD8^+^ splenocytes. (F) Representative FACS plots of CD3 and CD1d‐tetramer staining for thymocytes and splenocytes. (G, H) Percentages and numbers of thymic iNKT cells from WT and Pbrm1 KO mice. (I, J) Percentages and numbers of iNKT cells in the spleens from WT and Pbrm1 KO mice. The results shown are representative of three independent experiments. ^**^ *p* < 0.01. Data were shown as Mean ± SD

To investigate the role of Pbrm1 in the development of T‐cell lineages, we generated T cell‐specific Pbrm1 knockout mice by crossing Pbrm1 flox mice with LckCre transgenic strain. In consistent with previous studies,^38^ there were no developmental abnormalities in CD4^+^ and CD8^+^ T cells in the thymus and spleen after Pbrm1 deletion (Figures 1A–E and S1). Surprisingly, Pbrm1‐deficient mice exhibited a significantly decrease in iNKT cells in the spleen, rather than in the thymus compared to WT control (Figure 1F–J).

**FIGURE 2:**
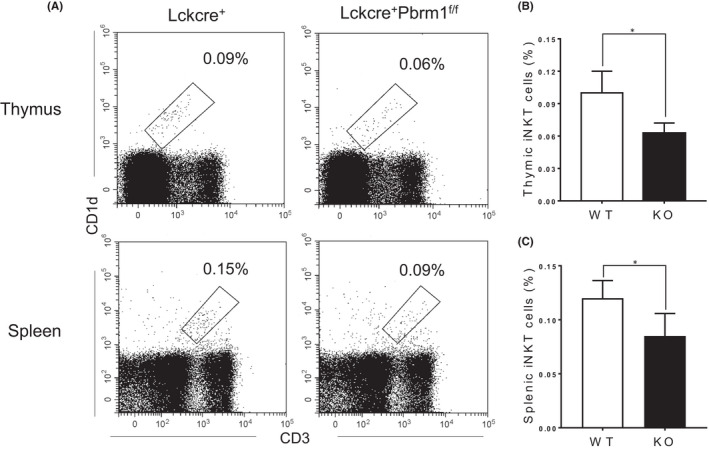
Pbrm1 is intrinsically required for the presence of iNKT cells in the periphery. CD45.1^+^ WT mice were irradiated with 7.5 Gy X‐ray and injected with lineage negative bone marrow cells from CD45.2^+^ Lckcre^+^Pbrm1^+/+^ (WT) or Lckcre^+^Pbrm1^f/f^ (KO), respectively. Recipient mice were sacrificed and analyzed 7 weeks after transplantation (*n* = 3). (A) Representative FACS plots showing CD3^+^ and CD1d‐tetramer^+^ iNKT cells from the thymi and spleens. (B) Statistical frequency and numbers of thymic iNKT cells from WT and Pbrm1 KO mice. (C) Statistical frequency and numbers of iNKT cells in the spleens from WT and Pbrm1 KO mice. The results shown are representative of three independent experiments. ^*^ *p* < 0.05. Data were shown as Mean ± SD

To further confirm the role of Pbrm1 in iNKT cells, we adoptively transferred bone marrow cells from either CD45.2^+^ Lckcre^+^Pbrm1^+/+^ (WT) or CD45.2^+^ Lckcre^+^Pbrm1^f/f^ (KO) mice into lethally irradiated (7.5 Gy) CD45.1^+^ congenic wild‐type recipients. Seven weeks after transplantation, thymocytes and splenocytes from recipients were harvested for the analysis. As expected, the percentage of CD4^+^ and CD8^+^ T cells in the thymus and spleen was not affected in the Pbrm1 deficient mice compared to WT mice (Figure S2). However, iNKT cells from mice received Pbrm1‐deficient donor cells showed a remarkable reduction of both the percentage and cell number in the thymus and spleen (Figure 2A–C). Together, we demonstrated that Pbrm1 is required for the population size of peripheral iNKT cells.

### 3.2 Pbrm1 deletion has no impact in the proliferation and survival of iNKT cells

**FIGURE 3:**
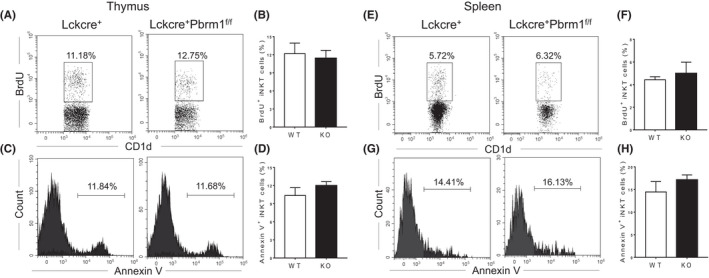
Pbrm1 deficiency has no effect on iNKT cell proliferation and survival. 1 mg of BrdU per mouse was i.p. injected to WT and Pbrm1 KO mice. 4 hours post injection, the thymi and spleens were harvested for FACS analysis (*n* = 3). (A) Representative FACS plots of CD1d‐tetramer and BrdU staining for thymic iNKT cells from WT and Pbrm1 KO mice. (B) Statistical frequency of BrdU^+^ cells in thymic iNKT cells. (C) Representative histograms of Annexin V staining of iNKT cells in the thymi. (D) Statistical frequency of Annexin V^+^ cells in thymic iNKT cells. (E) Representative FACS plots of CD1d‐tetramer and BrdU staining in the spleens. (F) Statistical percentage of BrdU^+^ cells in iNKT cells from the spleens. (G) Representative histograms of Annexin V staining of iNKT cells in the spleens. (H) Statistical percentage of Annexin V^+^ cells in iNKT cells from the spleens. The results shown are representative of three independent experiments

We then investigated whether the reduction of iNKT cells in the periphery was due to decreased homeostatic proliferation or increased cell death by performing BrdU incorporation and Annexin V staining assay in both thymocytes and splenocytes. Comparable percentage of BrdU^+^ cells (Figure 3A,B,E,F) as well as unchanged Annexin V^+^ iNKT cells (Figure 3C,D,G,H) were present in iNKT cells from the thymus and spleen in both Pbrm1 KO and WT mice. Consistently, similar proliferation and apoptosis between WT and Pbrm1 deficient iNKT cells were observed under α‐galactosylceramide (α‐GalCer) stimulation *in vitro* for 48 h (Figure S3). Thus, the reduction of iNKT cells in Pbrm1 KO mice is not due to the impairment of cell proliferation and survival.

### 3.3 Loss of Pbrm1 caused a differentiation block at stage 1 iNKT cells

**FIGURE 4:**
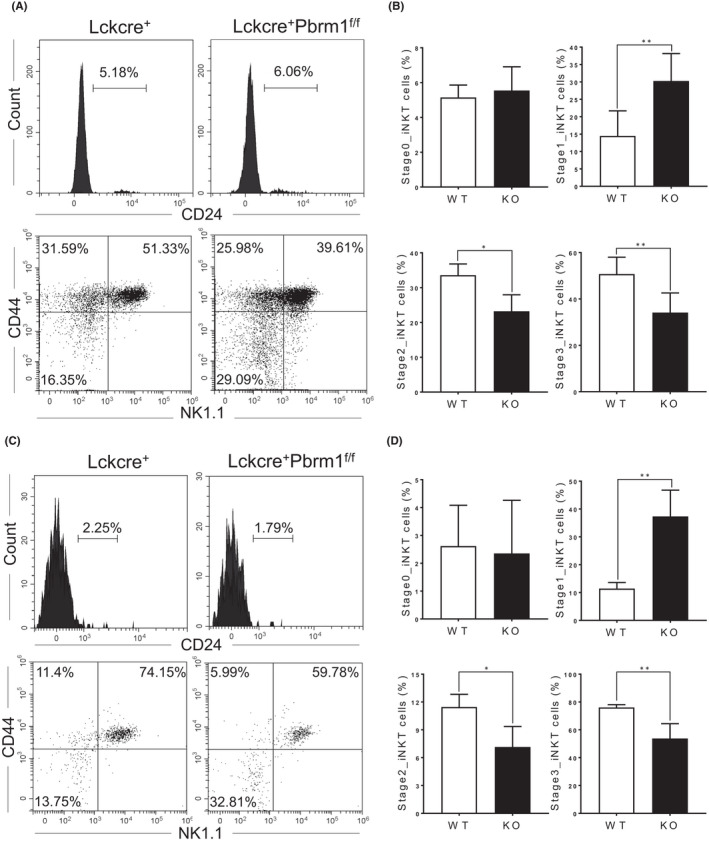
Effect of Pbrm1 deletion on developmental stages of iNKT cells. (A) Representative FACS plots showing developmental stages of iNKT cells from the the thymi. The developmental stages of thymic iNKT cells were analyzed based on the expression of CD24, CD44 and NK1.1. The upper plots are gated on CD1d‐tetramer^+^ cells and the lower plots are gated on CD24^−^CD1d‐tetramer^+^ cells. (B) Statistical frequency of iNKT cells at different developmental stages for (A). (C) Representative FACS plot showing developmental stages of iNKT cells from the thymi of mixed bone marrow chimeric mice as described in Figure 2. (D) Statistical frequency of iNKT cells at the indicated developmental stages for (C). *n* = 3 for each group. The results shown are representative of three independent experiments. ^*^ *p* < 0.05; ^**^ *p* < 0.01. Data were shown as Mean ± SD

We next sought to determine whether the decrease of iNKT cells was caused by the developmental retardation. Among the well‐defined signature genes for iNKT cell differentiation, we analyzed the expression of CD24, CD44 and NK1.1 in CD3^+^CD1d^+^ thymocytes. The results showed that Pbrm1 KO mice had similar percentage of CD24^−^ matured iNKT cells, nevertheless an increase of iNKT cell percentage at stag1 and a decrease of iNKT cells at both stage 2 and stage 3 in Pbrm1 KO mice in comparison to WT mice (Figure 4A,B). Consistently, as shown in Figure 4C,D, the dysfunction of iNKT cells in Pbrm1‐deficient mice was further confirmed in bone marrow chimera mice. Together, the reduced number of iNKT cells in Pbrm1 KO mice is most likely due to a developmental block from stage 1 to stage 2/3.

### 3.4 Pbrm1 is required for RORγt expressing iNKT17 cell differentiation

**FIGURE 5:**
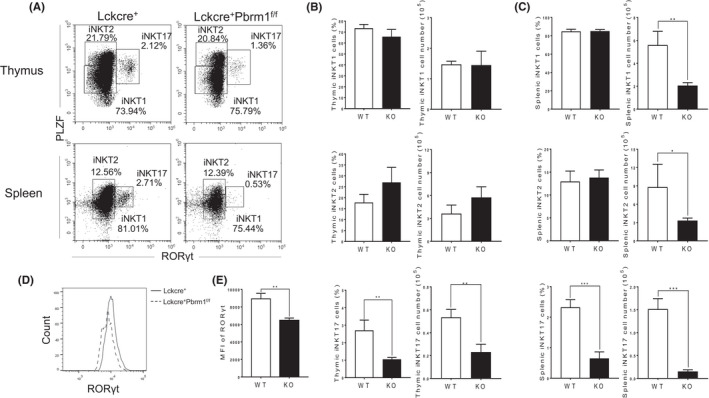
Pbrm1 deletion impairs iNKT17 cell differentiation, but not iNKT1 and iNKT2 generation. Eight‐week‐old Lckcre^+^Pbrm1^+/+^ (WT) and Lckcre^+^Pbrm1^f/f^ (KO) mice were analyzed for iNKT cells differentiation in both thymus and spleen by flow cytometry (*n* = 3). (A) PLZF and RORγt staining of thymic and splenic iNKT cells. iNKT cell effector lineages were in the indicated FACS plot. (B) Percentages and cell numbers of iNKT effector lineages in the thymi. (C) Percentages and cell numbers of iNKT effector lineages in the spleens. (D) The overlay histogram of RORγt expression in thymic iNKT17 cells from WT and Pbrm1 KO mice. (E) Mean fluorescence intensity (MFI) of RORγt expression in thymic iNKT17 cells from WT and Pbrm1 KO mice. The results shown are representative of three independent experiments. ^*^ *p* < 0.05; ^**^ *p* < 0.01; ^***^ *p* < 0.001. Data were shown as Mean ± SD

Functionally, the mature iNKT cells are categorized into different effector subsets according to unique transcription factor expression and cytokine production. We then aim to explore whether Pbrm1 also participates in the effector iNKT cell differentiation. Flow cytometry analysis showed that the percentage and total cell number of RORγt^+^ iNKT cells were significantly decreased in the thymus (Figure 5A,B) and even strikingly reduced in the spleen in Pbrm1 KO mice compared to WT mice (Figure 5A,C), whereas iNKT1 and iNKT2 cells remained unchanged in those mice. Moreover, Pbrm1‐deficient iNKT17 cells exhibited a significant decrease of RORγt expression in the thymus (Figure 5D,E). Taken together, these data demonstrated that loss of Pbrm1 specifically impaired RORγt expression and iNKT17 cell differentiation without affecting iNKT1 and iNKT2 cell populations.

## 4 DISCUSSION

In the current study, we reveal an intrinsic role of Pbrm1, as a subunit of SWI/SNF complex, in regulating iNKT cell development and differentiation. Pbrm1 deletion not only affected the developmental progression of thymic iNKT cells, but also impaired the effector differentiation of iNKT17 cells.

The SWI/SNF chromatin remodeler family integrates multiple components to execute biological functions, and loss of different components may lead to the same or diverse defect. So far, the data from our and other groups demonstrate that loss of either Pbrm1 or Brg1 does not impact αβ T cell development in the thymus and their homeostasis in the periphery.^38^, ^44^, ^45^ Brg1 regulates Th1 or Th2 differentiation under certain conditions,^38^ while Pbrm1 affects Treg cells maintenance and their immunosuppressive function.^32^ Here we uncover a novel regulation network that Pbrm1 intrinsically controls iNKT cell development and effector differentiation. Therefore, our study may hopefully pave the way for future studies of different SWI/SNF components in multiple aspects or immune cell population.

The pool size of peripheral iNKT cells is determined by thymocyte input, homeostatic proliferation and survival. Brg1, as the core subunit of SWI/SNF complex, has been shown to participate in the inhibition of immature thymocyte apoptosis through maintaining the Bcl‐xl expression.^39^ However, our data demonstrated that Pbrm1 deficient iNKT cells exhibited unaffected proliferation and cell survival evidenced by comparable BrdU incorporation and Annexin V staining. In fact, we found that Pbrm1 deficiency intrinsically impaired the transition from stage 1 to stage 2 and stage 3 during iNKT cell development in the thymus. As iNKT cells at stage 2 and stage 3 are capable to migrate to the periphery,^46^ our data support the hypothesis that the reduction of iNKTs in the periphery is attributed to the developmental retardation and a decrease of thymic output. It will be interesting to investigate how Pbrm1 controls iNKT cell development mechanistically in the future.

The distinct effector subsets of iNKT cells endow their potent protective immunity and immune tolerance.^47^ It was reported that SRG3, as a components of SWI/SNF complex could assist Th17 cell differentiation through collaboration with RORγt.^48^ Consistently, our data showed that iNKT17 subset, but not iNKT1 and iNKT2 cells, was reduced in Pbrm1 KO mice according to the PLZF and RORγt expression. Future work will explore how the complex or Pbrm1 alone affects RORγt expression and the function of iNKT cells in the animal model.

Taken together, our study reveals a crucial role for Pbrm1 in maintaining the pool size of iNKT cells in the periphery. Pbrm1 ensures the stage transition during thymic iNKT cell development and proper numbers into the periphery. Moreover, Pbrm1 is required for the presence of iNKT17 cells in both thymus and periphery. Our findings highlight the importance of Pbrm1 in iNKT cell development and effector differentiation. Further studies on the mechanisms of how Pbrm1 regulating those aspects in iNKT cells should be pursued.

## AUTHOR CONTRIBUTIONS

**Xin Wang:** Investigation (equal). **Lei Lei:** Investigation (equal); writing – original draft (equal). **Yanhong Su:** Investigation (equal). **Jun Liu:** Investigation (supporting). **Ning Yuan:** Investigation (supporting). **Yang Gao:** Investigation (supporting). **Xiaofeng Yang:** Investigation (supporting). **Chenming Sun:** Conceptualization (equal); writing – original draft (equal); writing – review and editing (equal). **Bin Ning:** Writing – review and editing (equal). **Baojun Zhang:** Conceptualization (equal); funding acquisition (equal); project administration (lead); writing – review and editing (equal).

## CONFLICT OF INTEREST

The authors declare no conflict of interests.

## Supporting information


Figure S1

Figure S2

Figure S3

## Data Availability

All data and models during the study are available in the submitted article.
