# Supplementary figures and images for: Pbrm1 intrinsically controls the development and effector differentiation of iNKT cells
Source: J Cell Mol Med. 2022 Jun 29;26(15):4268–76. doi: 10.1111/jcmm.17445 (PMC9344823; doi:10.1111/jcmm.17445)

Fig. S1 Cell numbers of CD4+ and CD8+ T cells in thymi and spleens from WT and Pbrm1 KO mice

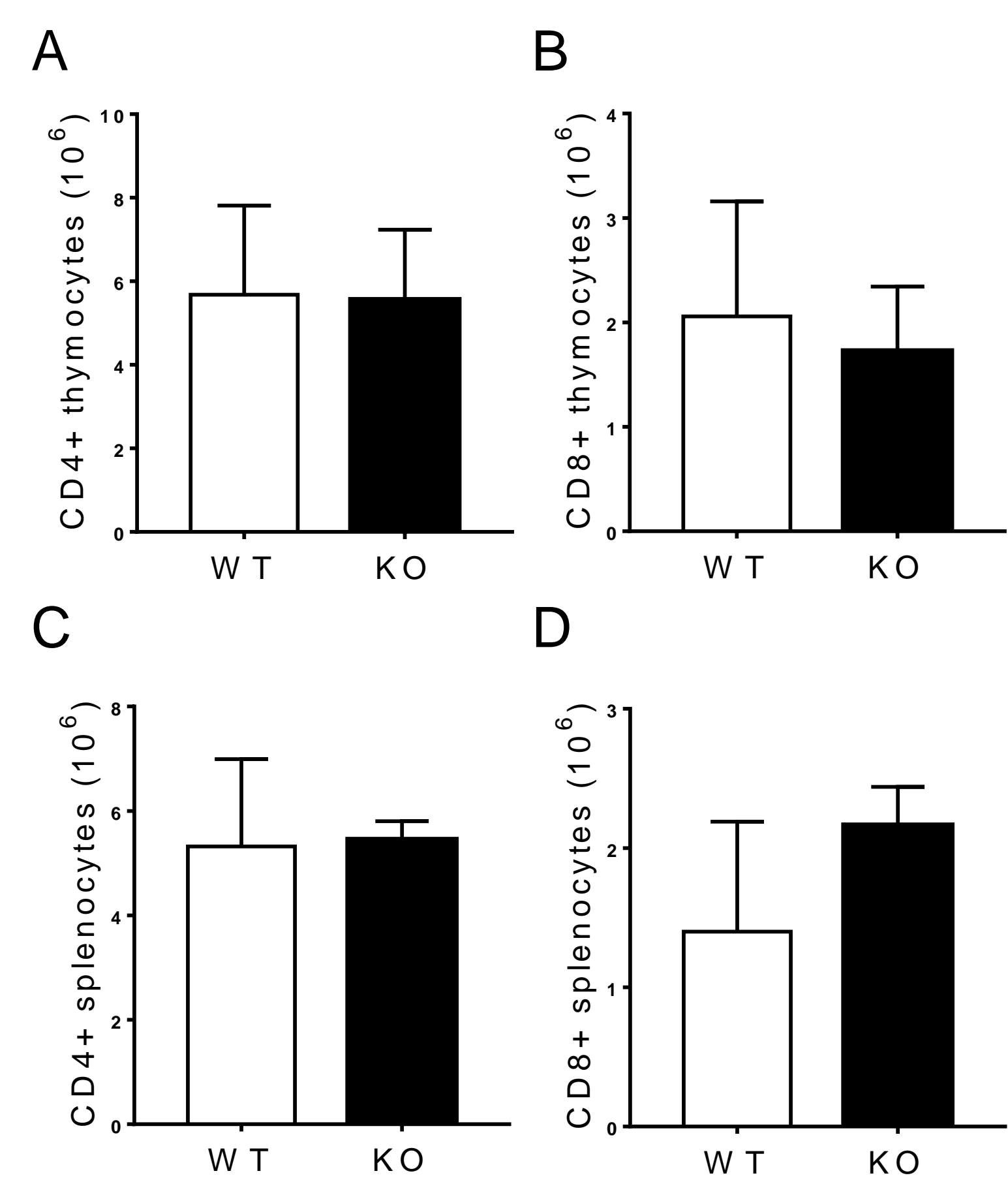

Supplement: Supplementary file 1 — Figure S1 [file JCMM-26-4268-s003.pdf]

Fig. S2 Percentages of CD4+ and CD8+ T cells in thymi and spleens of bone marrow transfer model

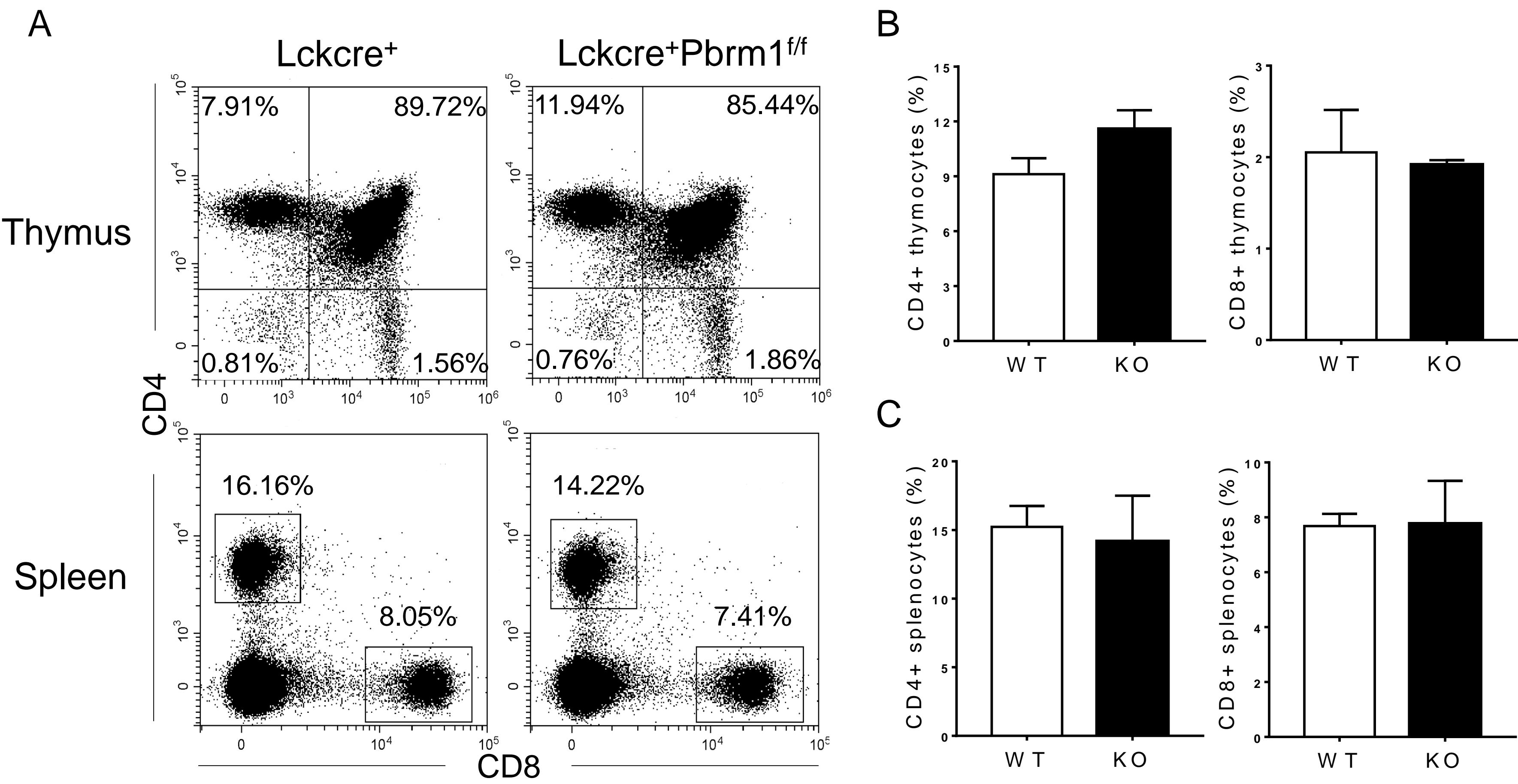

Supplement: Supplementary file 2 — Figure S2 [file JCMM-26-4268-s001.pdf]
