# Supplementary material for: Pbrm1 intrinsically controls the development and effector differentiation of iNKT cells
Source: J Cell Mol Med. 2022 Jun 29;26(15):4268–76. doi: 10.1111/jcmm.17445 (PMC9344823; doi:10.1111/jcmm.17445)

Fig. S3 Effect of Pbrm1 deficiency on iNKT cell proliferation and apoptosis after α-galactosylceramide stimulation

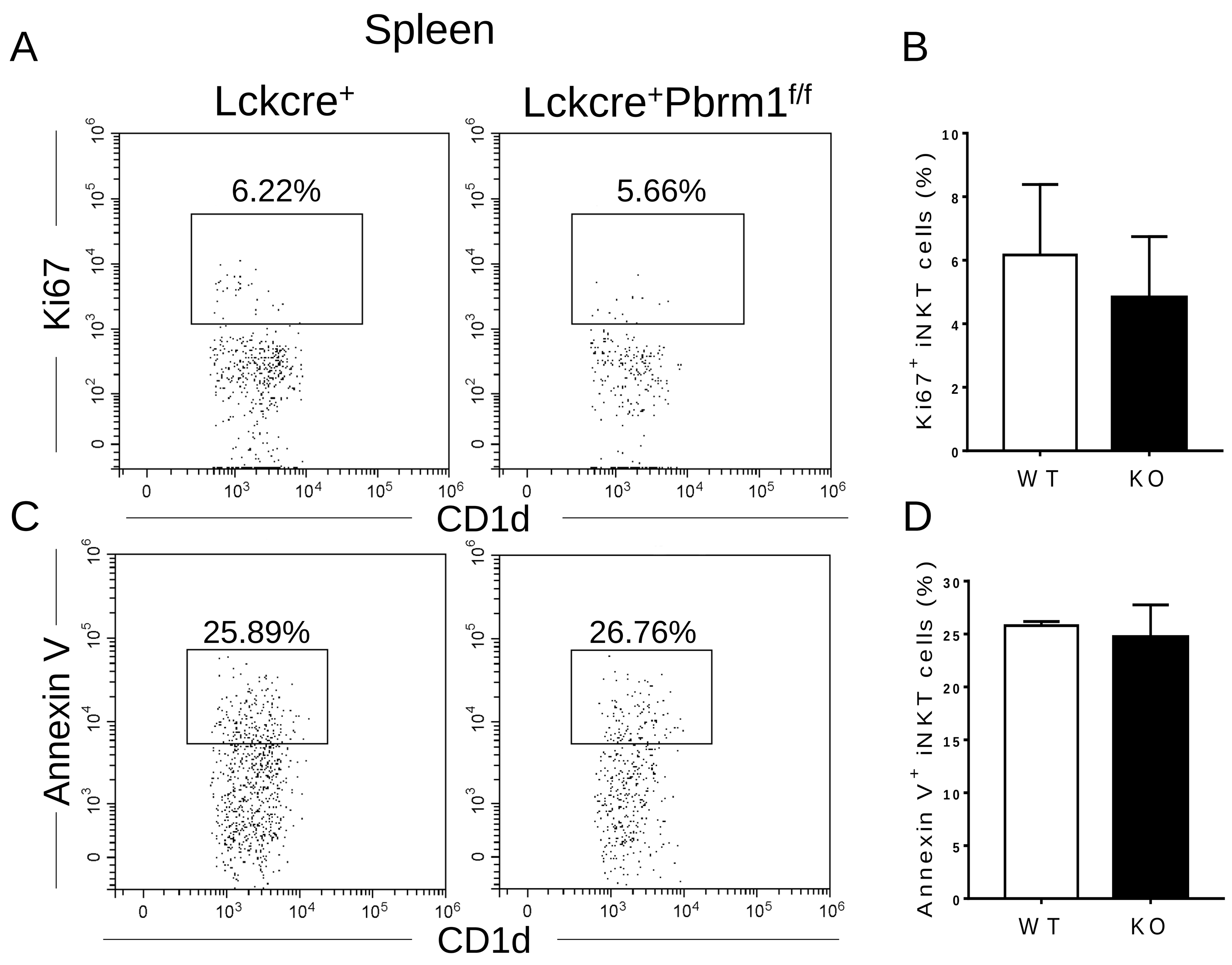

Supplement: Supplementary file 3 — Figure S3 [file JCMM-26-4268-s002.pdf]
